# Bacteriological quality and physicochemical analysis of the Kalte River at Wolaita Sodo Town, southern Ethiopia

**DOI:** 10.1186/s13104-024-06854-0

**Published:** 2024-07-09

**Authors:** Krishna Moorthy Sivalingam, Israel Solomon Choramo, Eyob Chukalo Chutulo

**Affiliations:** 1 Department of Biology, College of Natural and Computational Sciences, Wolaita Sodo University, P.O. Box 138, Wolaita Sodo, Ethiopia; 2 Department of Biology, College of Natural and Computational Sciences, Dilla University, P.O. Box 419, Dilla, Ethiopia

**Keywords:** Bacteriological analysis, Kalte River, Physicochemical analysis, Water quality, Coliform, Wolaita Sodo Town

## Abstract

**Objective:**

The current research aimed to investigate the physicochemical and bacteriological quality status of the Kalte River in Wolaita Sodo Town, southern Ethiopia.

**Methods:**

A total of 42 water samples were collected using sterile glass bottles from three different river sites: Damota (upstream), Kera (midstream), and Gututo (downstream). All the water samples were examined for the presence of heterotrophic bacteria, total coliform and fecal coliform using direct plate count method and membrane filtration method. Standard methods suggested by American water works association were used to analysis the physicochemical parameters of the water samples.

**Results:**

The results revealed that the total heterotrophic bacteria, total coliform, and fecal coliform count ranged from 8.9 to 12.6 × 10^4^ cfu/ml, 7.5–11.3 × 10^2^ cfu/ml and 5.7–9.7 × 10^4^ cfu/ml, respectively. The bacterial count results indicated that the river water crossed the WHO-recommended limit of potable water. *Escherichia coli, Pseudomonas aeruginosa, Staphylococcus aureus, Salmonella, and Shigella* species were the common bacterial pathogens isolated from river water samples. The results of the physicochemical analysis revealed that some of the parameters Biological Oxygen Demand (BOD), Chemical Oxygen Demand (COD), and turbidity exceeded the maximum permissible limits of WHO and other parameters were below the WHO permissible limits.

**Conclusion:**

Therefore, the presence of bacterial pathogens, fecal coliform indicators, and some physicochemical parameters of the Kalte River exceeding the recommended limits may expose users of the river water to the risk of infection.

**Supplementary Information:**

The online version contains supplementary material available at 10.1186/s13104-024-06854-0.

## Introduction

Rivers are one of the most abundant and readily available sources of fresh water for all living things, including humans [16]. Unfortunately, most of the rivers are polluted by different factors, such as the disposal of untreated industrial waste, sewage waste, and the overabundance of human activities, which affect the physio-chemical and bacteriological characteristics of the river [4].

Using contaminated drinking water and having poor sanitation are the major causes of these diarrhea cases. In poor nations, diarrhea mortality and morbidity are exacerbated by a lack of good water and sanitation. Over 800 million people are affected in Africa and Asia where access to clean water is a serious issue [18]. In Ethiopia, with a population of 75 million, more than half of the population has no access to safe water and it is estimated that approximately 35 million people do not have access to sanitation services [10]. In Ethiopia, more than 60% of transmissible diseases are due to unsafe and inadequate water supplies and poor hygiene and sanitation practices [15].

The Kalte River is one of several rivers in Ethiopia that receives solid and liquid wastes from domestic settlements, and hence understanding the status of the water quality is necessary by monitoring the physico-chemical and bacteriological analyses that enable us to understand the health risks associated with Kalte River water. However, no previous studies have investigated the physicochemical and bacteriological parameters that can indicate the quality of the water of Kalte River. Therefore, this study fills this gap by investigating the bacteriological quality analysis and physico-chemical parameters to understand the status of the water quality of the Kalte River.

## Materials and methods

### Description of the study area

**Fig. 1:**
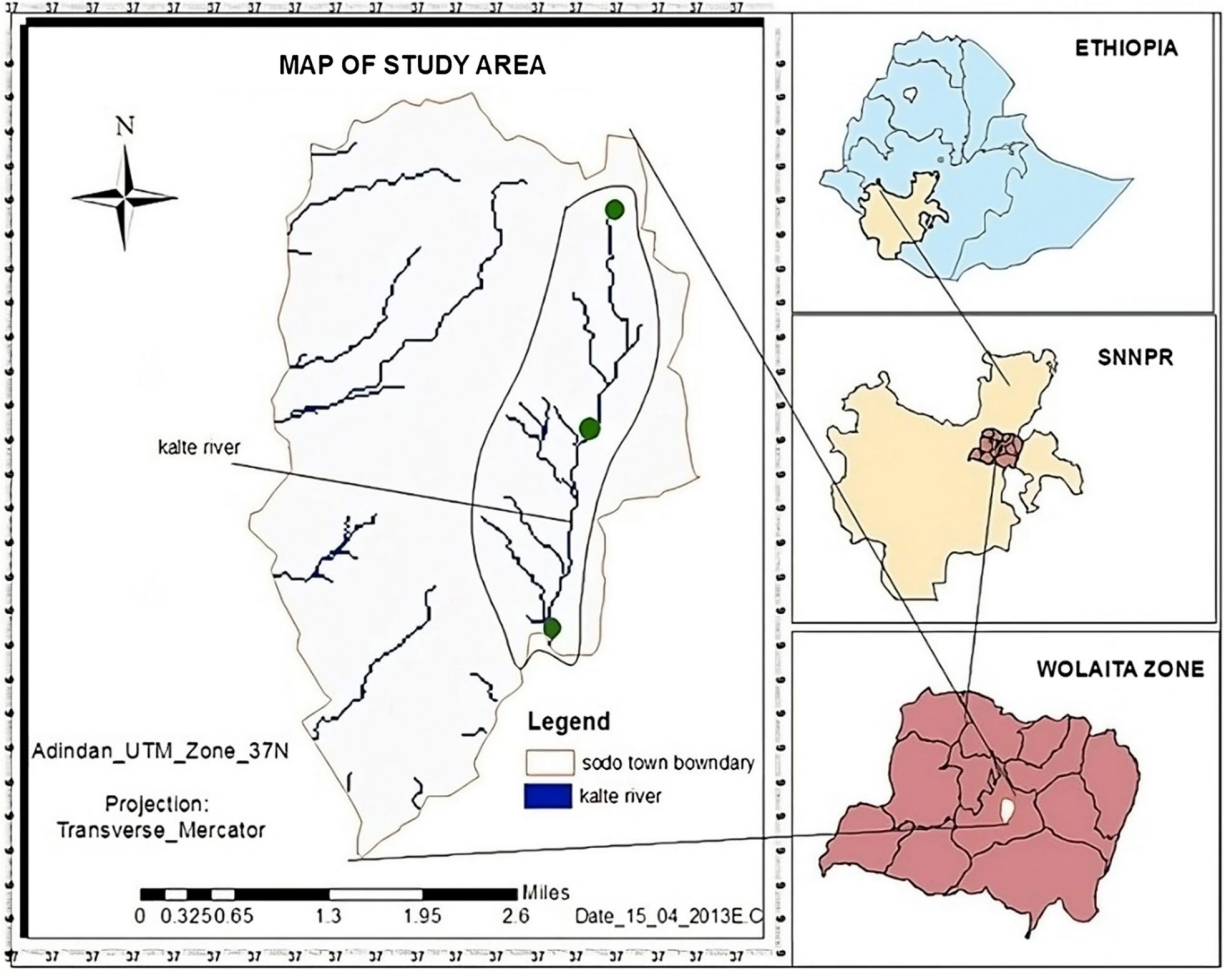
Map of Study areas (*Source:* GIS Ethiopia, 2016)

The study was conducted on the Kalte River in Sodo Town, southern Ethiopia (Fig. 1). It is located 340 km and 160 km away from Addis Ababa and Hawassa, respectively [20]. The location of the study area is in Markato Sub-city, Fana Kebele, Sodo Town, and entirely relies on Kalte Rivers. The Kalte River drains from the highland areas of Damota Mountain, with main small drainage systems in that the middle part of the catchments drains from Kalte womba to Sorfela locality.

### Sampling sites and sample collection

In total, forty-two (42) samples were collected from three sampling sites (Damota/Upstream, Kera/Midstream, and Gututo/Downstream). The physical parameters like temperature, turbidity, total dissolved solids, conductivity and pH were analysed onsite using portable thermometer, turbidity and pH meters. At each sampling station, fourteen (14) water samples [1] were purposively collected for bacteriological and physicochemical analysis using clean sterile containers (Fig. S1). All samples were appropriately labeled and stored in an ice box for transport to the Post Graduate Microbiology Laboratory at Wolaita Sodo University and analyzed within 6 h of sample collection according to WHO guidelines for sample collection [35].

### Enumeration of total heterotrophic bacteria

The heterotrophic plate count was carried out using plate count agar as described by Aliyu et al. [4]. The number of colonies forming units was counted, and the values were multiplied by the dilution factor to obtain the actual microbial levels (Fig. S2).

### Enumeration of fecal coliform and total coliform

A volume of 0.1 ml of serial diluted sample was aseptically transferred to the center of a prepared Eosin Methylene Blue (E.M.B) Agar and incubated. Lactose-fermenting colonies (Fig. S2) formed were counted as fecal coliform in cfu/ml, and the value was multiplied by the dilution factor to obtain the actual level of the bacteria in each of the collected water samples [4]. The membrane filtration technique was employed in the evaluation of the total coliform count. The membrane filter with 47 mm diameter, with 0.45 μm pore size (Merck Millipore, USA) was used to filter the river water samples according to Akubuenyi et al. [3]. After filtration, transfer the membrane filter and rolled over the surface of a Petri dish containing Endo Agar Base. Colonies that were dark red, mucoid, had a dark center, or (more typically) produced a metallic sheen were counted (Fig. S2) and counted as described by Forster and Pinedo, [12].

### Isolation and identification of selected bacteria from river water samples

A volume of one milliliter (1 ml) of river water sample was mixed with 9 ml of peptone water as pre-enrichment and incubated at 37 °C for 24 h and then streaked on different selective media such as Endo agar base for *E. coli*, mannitol salt agar for *S. aureus*, Salmonella-Shigella agar for *Salmonella* sp. and *Shigella* sp. and CLED agar for *P. aeruginosa* [4, 21]. All isolated bacterial cultures were identified by several standard biochemical tests referred with Bergey’s Manual of Determinative Bacteriology [9].

### Physico-chemical analysis of river water samples

In this study, the water physicochemical parameters were determined following the Standard Methods for the Examination of Water and Wastewaters [7].

### Data analysis

The results were analyzed using Statistical Package for Social Sciences (SPSS) software version 21 and Microsoft Office Excel. The data obtained were subjected to analysis for means, standard deviation, and significance between the means at the 5% probability level. The data were analyzed using single factor analysis of variance (ANOVA); thereafter, individual means were compared using post-hoc comparisons and were assessed using Dunnett’s T3.

## Results and discussion

### Bacteriological analysis of the Kalte River of Wolaita Sodo

The study results in the Table 1 indicated that all samples were collected from the rivers severely contaminated with total heterotrophic, total coliform, and fecal coliform bacteria and exceeded the standard guidelines for drinking water quality set by the WHO [36]. This could be attributed to the discharge of domestic and agricultural wastes as well as human excreta/wastes into the Kalte River. According to a report by Izah and Angaye [17], domestic waste and sewage are discharged into most surface water by communities aligning rivers. The present study is in line with the reports of Sebsibe et al. [28], Hailu [15], Gadiso et al. [13], Birtukan et al. [8], and Amenu et al. [5] which indicated that all water samples collected were positive for total coliforms and fecal coliforms. The findings of this study are also comparable to the reports of Akubuenyi et al. [3], Seiyaboh et al. [30], Seiyaboh and Kolawole [29], and Omoigberale et al. [24].

**Table 1:** Quantitative analysis of bacterial contamination in the Kalte River of Wolaita Sodo Town

Sample site	Total heterotrophic (CFU/ml)	Fecal coliform (CFU/ml)	Total coliform (CFU/ml)
Damota	8.9 ± 0.7 × 10^4^	5.7 ± 1.1 × 10^4^	7.5 ± 0.78 × 10^2^
Kera	12.6 ± 0.78 × 10^4^	9.7 ± 0.99 × 10^4^	11.3 ± 0.84 × 10^2^
Gututo	10.9 ± 0.71 × 10^4^	8.1 ± 0.68 × 10^4^	9.6 ± 0.76 × 10^2^

Data are expressed as the mean ± standard deviation; the mean difference is significant at the 0.05 level (P < 0.05)

### Isolation and identification of bacterial pathogens from water samples of the Kalte River

In the present study, five bacterial pathogens i.e., *E. coli, Salmonella* species, *Shigella* species, *S. aureus,* and *P. aeruginosa* were isolated from river water samples collected from different sites of the Kalte River of Wolaita Sodo by using selective media. All the abovementioned bacterial pathogens were selected as suspected forms from selective media based on the color of colony morphology (Table 2, Table S1).

**Table 2:** Isolation of bacterial pathogens based on colony morphology using selective media

Isolates	Selective Agar	Colony morphology
*Escherichia coli*	Endo Agar Base	Pink to a rose-red, green metallic sheen -Lactose Fermenter
*Salmonella* species	Salmonella Shigella Agar	Colonies with large black centers
*Shigella* species	Salmonella Shigella Agar	Colorless colonies
*Staphylococcus aureus*	Mannitol salt agar (MSA)	Golden yellow colonies with yellow color zone around the colonies
*Pseudomonas aeruginosa*	CLED agar	Bluish Green colonies with typical mattered surface and rough periphery

Colony morphology is one of the important preliminary examinations to isolate appropriate bacterial cultures. Similarly, a study conducted by Krishna Moorthy and Subramaniyan, [21] revealed the isolation and identification of common bacterial pathogens using selective media from well water samples. All the bacterial cultures isolated based on colony morphological characteristics were further confirmed by biochemical characterization using different biochemical tests. The biochemical test results are presented in the form of tables (Tables 3, 4).

**Table 3:** Identification of bacterial pathogens isolated from water samples of Kalte River, Wolaita Sodo Town

S. No	Name of test	Response of *E. coli*	Response of *Salmonella* spp.	Response of *Shigella* spp.
1	Gram’s staining	Gram-negative, Rods	Gram-negative, Rods	Gram-negative Rods
2	Motility	Positive	Positive	Negative
3	Indole production	Positive	Negative	Negative
4	Sulfide production	Negative	Positive	Negative
5	Methyl red	Positive	Positive	Positive
6	Urease production	Negative	Negative	Negative
7	Citrate utilization	Negative	Positive	Negative
8	Catalase test	Positive	Positive	Positive
9	Coagulase test	Negative	Negative	Negative

**Table 4:** Identification of bacterial pathogens isolated from water samples of Kalte River, Wolaita Sodo Town

S. no	Name of test	Response of *P. aeruginosa*	Response of *S. aureus*
1	Gram’s staining	Gram-negative, Rods	Gram-positive, Cocci
2	Motility	Positive	Negative
3	Indole production	Negative	Negative
4	Sulphide production	Negative	Negative
5	Methyl red	Negative	Positive
6	Urease production	Negative	Positive
7	Citrate utilization	Positive	Positive
8	Catalase test	Positive	Positive
9	Coagulase test	Negative	Positive

### Prevalence of common bacterial pathogens in the water samples collected from the Kalte River

**Fig. 2:**
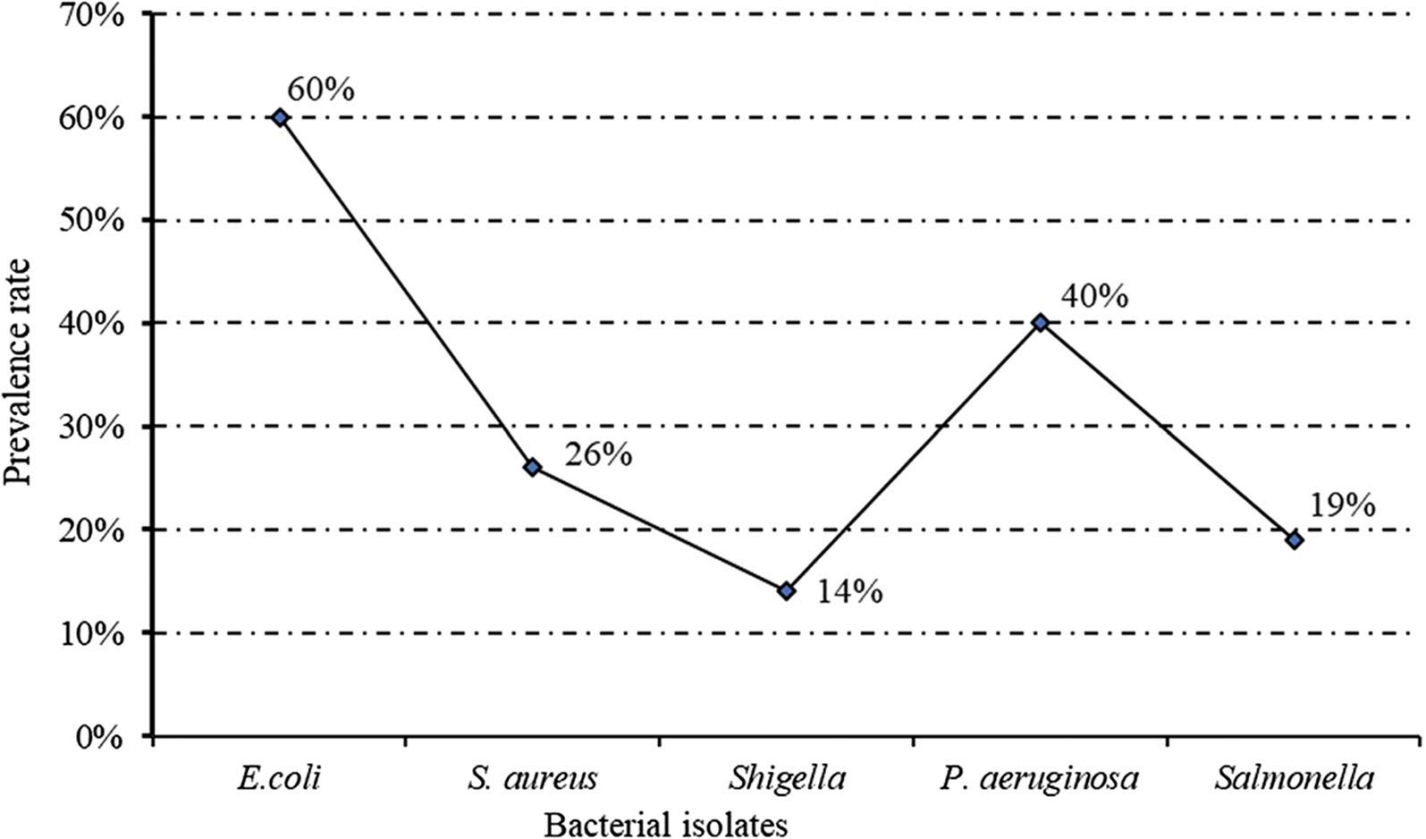
Prevalence rate of human pathogenic bacteria in river water samples collected from the Kalte River of Wolaita Sodo, Ethiopia

According to this study, the dominant organism among the isolates was *E. coli,* which was found to be positive in 25 water samples with a 60% prevalence, followed by *P. aeruginosa,* which was present in 21 water samples with a 40% prevalence. The lowest prevalence rate was *Shigella* spp., with 14% (Fig. 2). Similarly, a study reported by Abhishek et al. [1] reveals that the occurrence of *E. coli* was 61%, Salmonella 25%, S. *aureus* 14%, and *P. aeruginosa* 53% as 22, 9, 5, and 19 samples out of 36 water samples were found contaminated, respectively. In a similar study, the most isolated organisms from water sources included *E. coli* (22.7%), *Salmonella* spp. (13.3%), *Shigella* spp. (19.3%), *Proteus* spp. (18.5%), *Klebsiella* spp. (19.3%), and *P. aeruginosa* (4.2%) [23]. The various bacterial contaminants of water have been reported by Wekulo et al. [34], Akubuenyi et al. [3], Seiyaboh et al. [30] and Seiyaboh and Kolawole [29]. Terfasa et al. [32] reported that bacterial pathogens such as *Staphylococcus* spp*., E. coli, Shigella* spp*., Bacillus* spp*., Salmonella* spp*.* frequently found in the Wabe River of Ethiopia. In a related study, Gebrewahd et al. [14] reported the presence of *E. coli*, *Salmonella* spp., *Shigella* spp., *Yersinia*, *Campylobacter*, *Legionella*, and *Pseudomonas* as the most common isolates in their study from potable water sources. These bacteria are microbes of public health importance. The bacterial isolate mainly belongs to the family Enterobacteriaceae, which is known to consist of several pathogenic bacteria [34]. *Salmonella* spp. and *E. coli* are considered food and waterborne pathogens, and *E. coli* is a good indicator of fecal contamination of water [2].

### Physico-chemical analysis of water samples collected from the Kalte River

The physicochemical analysis results are presented below in Tables 5, 6 and 7. The temperature is one of the most important ecological factors, and is closely related to latitude, altitude, and season [22]. The present study is in line with the reports of Ken-Onukuba et al. [19], Raji et al. [26], Terfasa et al. [32], and Tesfaye et al. [11].

**Table 5:** Physical parameters of the river water samples collected from the Kalte River

Sites	Parameters						
	Temp. (°C)	pH	Cond. µS/cm	TDS (mg/l)	TA (mg/l)	Turb. (NTU)	TH mg/l CaCO_3_
Damota	15.7 ± 1.2	7.67	42.75 ± 15.1	28.5 ± 11	102	8	90
Kera	20.9 ± 1.7	7.93	185.5 ± 9.5	125 ± 6.5	162	10	140
Gututo	22.8 ± 1.8	7.95	184.3 ± 48.5	123 ± 33	122	8	80

*Temp.* water temperature, *Cond.* Conductivity, *TDS* total dissolved solids, *Turb* turbidity, *TA* total alkalinity, *TH* total hardness

**Table 6:** Chemical oxygen demand (COD) and biological oxygen demand (BOD) of the river water samples collected from the Kalte River

Sites	BOD mg/l	COD mg/l
Damota	42.5	53
Kera	79.7	91
Gututo	59.6	82

**Table 7:** Chemical parameters of the river water samples collected from the Kalte River

Chemical parameters	Unit	Sample sites		
		Damota	Kera	Gututo
NH4^+^	mg/l	0.61	1.53	1.21
NO^2−^	mg/l	0.15	1.31	1.31
NO_3_^−^	mg/l	5.0	3.9	6.3
PO_4_^3−^	mg/l	0.18	0.31	1.95
HCO_3_^−^	mg/l	122	250	146
Na^+^	mg/l	11.0	8.5	28.2
K^+^	mg/l	3.1	12.0	4.0
Ca^+^	mg/l	8	28	24
Mg^+^	mg/l	17	17	4.9
Fe	mg/l	0.56	0.42	0.33
Cu^2+^	mg/l	0.22	0.07	0.8
Mn^2+^	mg/l	0.1	0.1	0.2
Cr^6+^	mg/l	0.02	0.03	0.02
F^−^	mg/l	0.08	0.20	0.19
Cl^−^	mg/l	< 10	< 10	< 10

pH is used to measure the intensity of acidity or alkalinity of water [27]. The pH values of the water samples in the study area ranged from 7.67 to 7.95 (Table 5). The recorded pH value of this study is comparable with other studies reported from different areas, where pH value ranged from 7.2 to 7.3 in the Wabe River of Wolkite town [32], 6.65–6.96 in the Jakara River of northwestern Nigeria [6] and the average value in the Hare River (7.8) and Kulfo River (8.4) in Arbaminch town of southern Ethiopia [33].

The TDS value observed in this study was below the WHO recommended limit (500 mg/l) [36]. The present study is in line with the reports Raj et al. [25] and Teshome et al. [33]. Hardness is a measure of how much calcium and magnesium is present in water [11]. The variations in total hardness may be because of decomposition and mineralization of organic materials [31]. The results of the present study indicate (Table 5) that the value of total hardiness was below the maximum permissible limit of drinking water standards (500 mg/l CaCO_3_) [36]. The result obtained in the present study is comparable with other studies reported by Tesfaye et al. [11], Raji et al. [26] and Ken-Onukuba et al. [19].

The values of BOD and COD observed in this study are presented in Table 6. The results of the present study when compared to the results of other similar studies of Ken-Onukuba et al. [19] and Tesfaye et al. [11], the COD and BOD records of Kalte River in Sodo Town were high. The higher BOD and COD in Kalte River might be due to the high nutrient concentration from domestic wastes and the presence of more organic load from urban settlements and slaughterhouse wastewater. High values of BOD and COD indicate that the Kalte River is unfit for human consumption.

## Conclusion

The present study indicated the detrimental impacts of anthropogenic activities on the water quality of the Kalte River. Slaughterhouse effluents, the wheat flour milling industry effluent, domestic sewage, wastes from cloth washing close to the river, and agricultural input are the major environmental pollutants deteriorating the river water quality. The pollutants increased bacterial contamination and significantly changed the physicochemical quality parameters of the Kalte River. The anthropogenic impact resulted in moderate and heavy pollution of Damota and Gututo sites, and Kera station, respectively.

The bacteriological profile of the river confirmed the presence of *S. aureus*, *E. coli*, *Salmonella*, and *Shigella* species together with the opportunistic *P. aeruginosa*. The high load of indicator organisms in the water samples of the study area suggested that the Kalte River water is polluted at a moderate level of contamination with faecal coliforms that potentially can threaten anyone consuming the water. The physicochemical parameters such as turbidity, BOD, and COD were above the maximum permissible limits of potable water at all sites. In conclusion, the Kalte River of Wolaita Sodo is polluted with agricultural, domestic, and industrial wastes as well as fecal contamination, which makes river water unsafe for human consumption and poses a risk of infection. Therefore, the government authority and regulatory body should take necessary intervention action to regulate domestic and industrial waste disposal as well as proper public awareness on the health and socioeconomic risks of microbial (fecal) contaminants is deemed necessary to maintain the quality and status of the Kalte River.

### Limitations

Due to lack of finance, the sampling strategy were restricted only in three different sites of the Kalte River. However, it was better to sample more sites of river. Since, this study conducted within April to June in dry season, it was not able to see Kalte River by seasonal variation in terms of water quality. This study focused on only limited to five different bacterial pathogens, due to lack of microbiological media and chemicals.

### Supplementary Information

Additional file 1.

## Data Availability

The author declares that all available data are reported in this paper.

## Declarations

### Declarations

All authors have read, understand, and have complied as applicable with the statement on “Ethical responsibilities of Authors” as found in the instructions for authors.
