# Supplementary material for: Bacteriological quality and physicochemical analysis of the Kalte River at Wolaita Sodo Town, southern Ethiopia
Source: BMC Res Notes. 2024 Jul 9;17:192. doi: 10.1186/s13104-024-06854-0 (PMC11234556; doi:10.1186/s13104-024-06854-0)
Supplement: Supplementary file 1 — Additional file 1. [file 13104_2024_6854_MOESM1_ESM.docx]

**SUPPLEMENTARY DATA**

**A. Damota site**

**B. Kera site**

**D. Collected water samples**

**C. Gututo site**


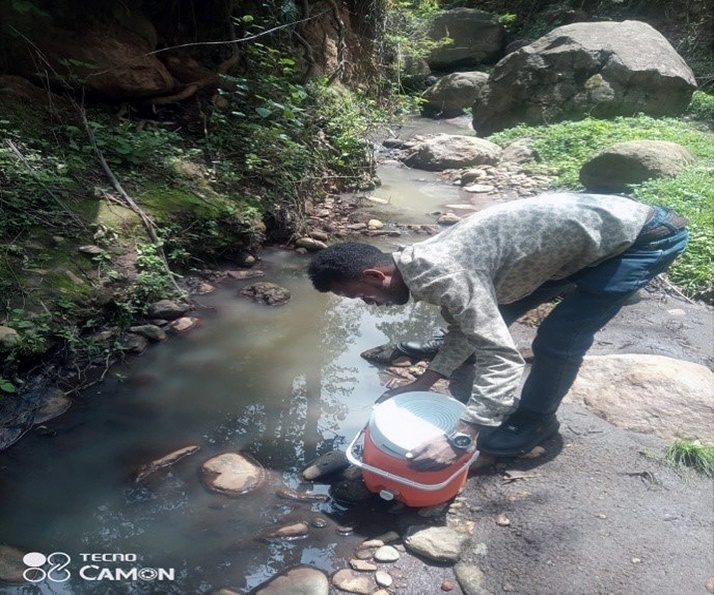

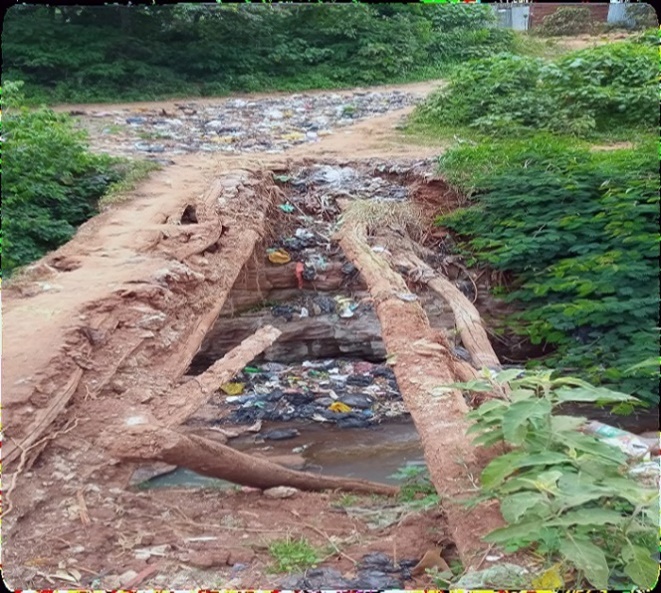

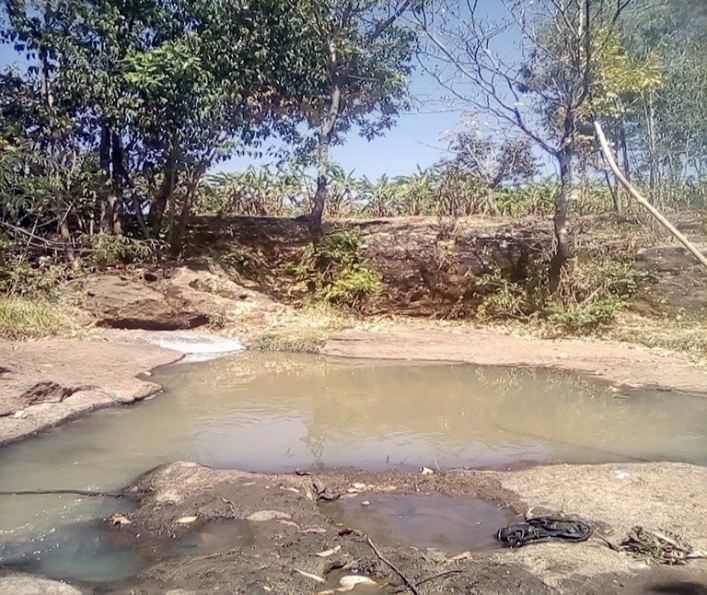

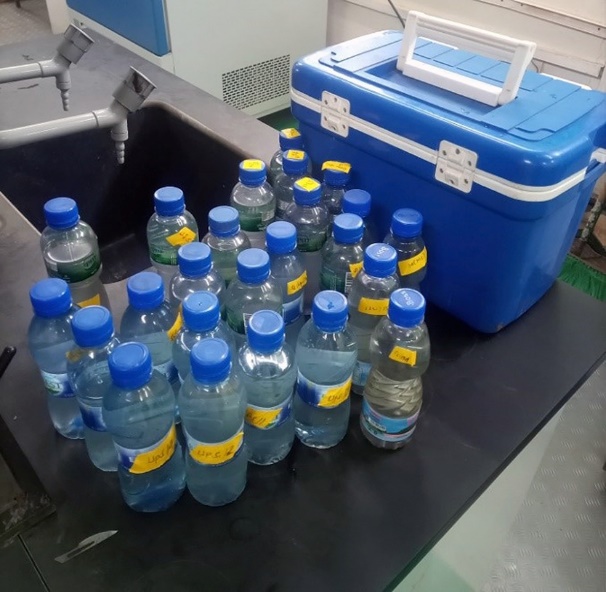


**Figure S1.** Sample collection from different sites of the Kalte River

**Total heterotrophic count (THC)**

**Fecal coliform count (FCC)**

**Total coliform count (FCC)**


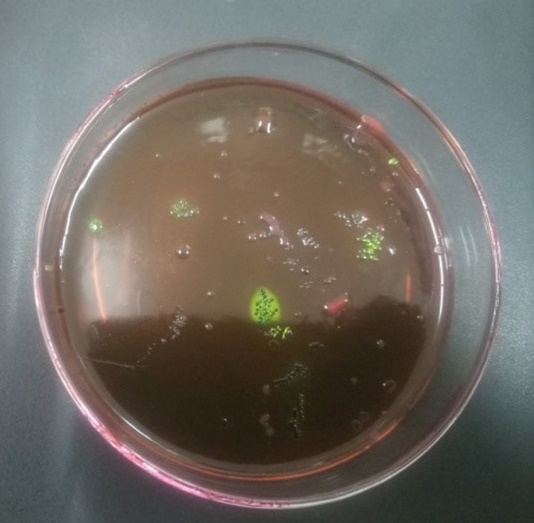

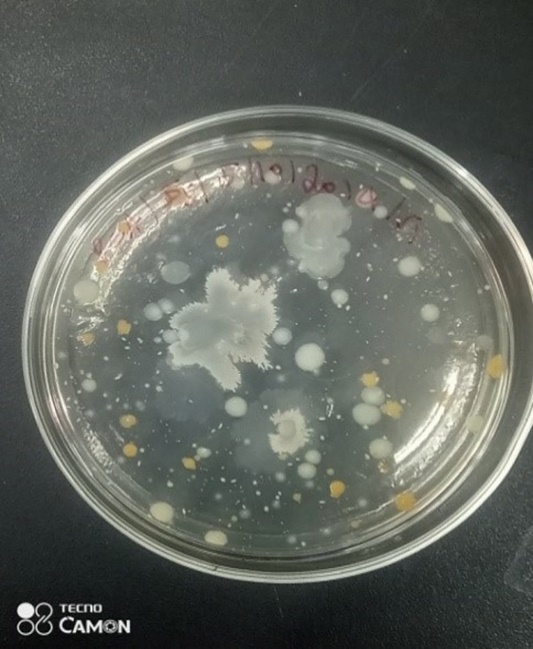

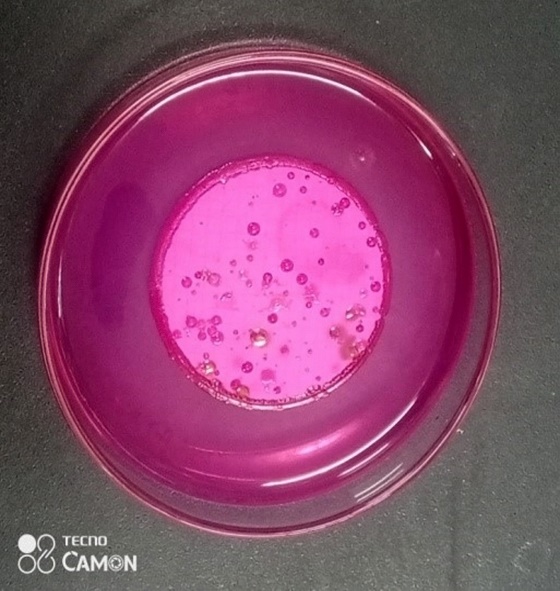


**Figure S2.** Analysis of bacteriological load in the water samples collected from the Kalte River

***Escherichia coli***

***Salmonella* species**

***Shigella* species**

***Staphylococcus aureus***

***Pseudomonas aeruginosa***


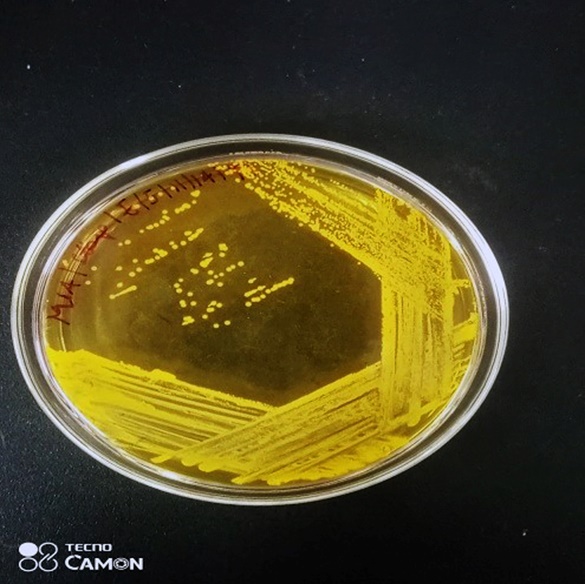

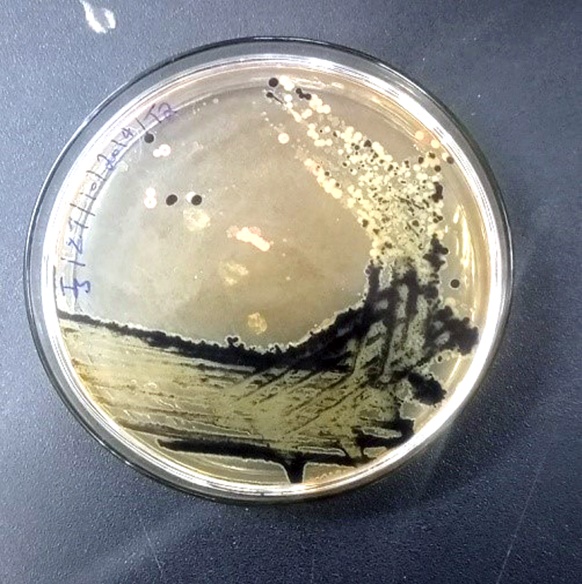

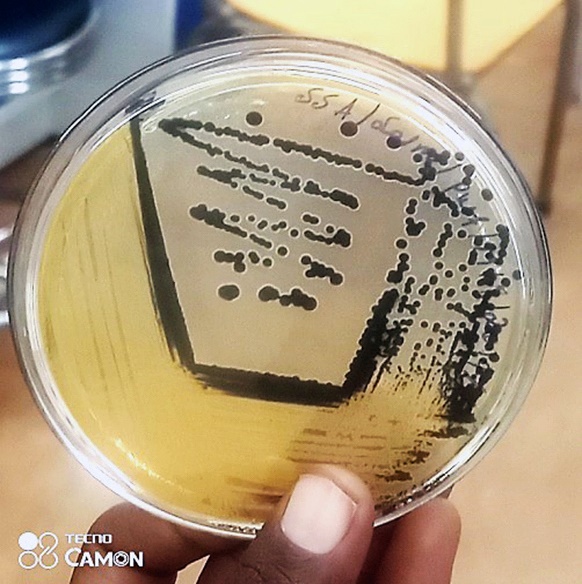

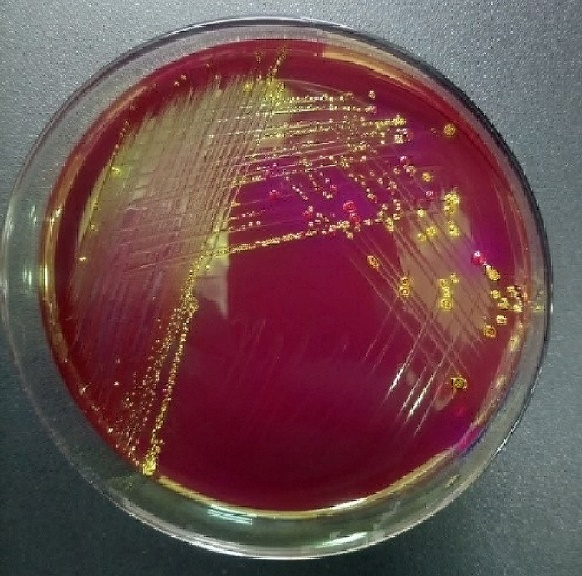

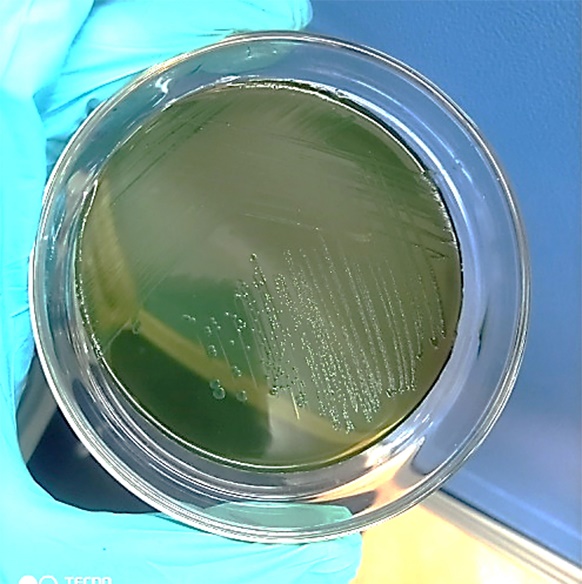


**Figure S3.** Isolation of different bacterial pathogens from the water samples of the Kalte River

**Urease Test:** A - *E. coli* (Negative); B – *S. aureus* (Positive); C – *Salmonella* spp. (Negative); D – *Shigella* spp. (Negative); E – *P. aeruginosa* (Negative)

**Citrate Utilization Test:** A – *S. aureus* (Positive); B – *Shigella* spp. (Negative); C – *P. aeruginosa* (Positive); D - *E. coli* (Negative); E – *Salmonella* spp. (Positive); F – Control


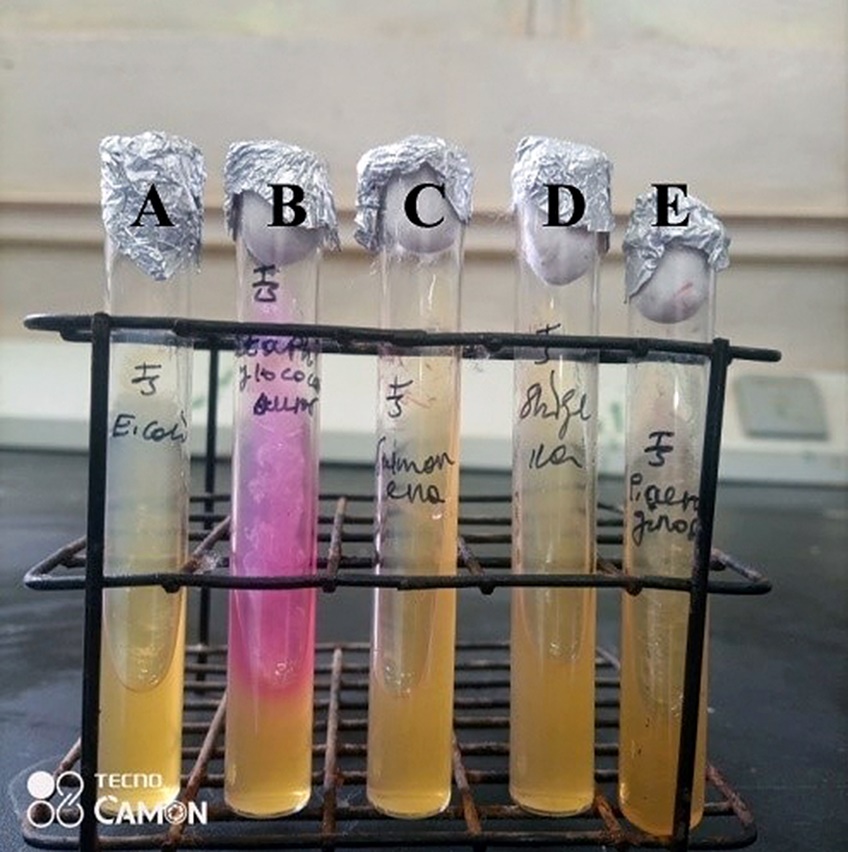

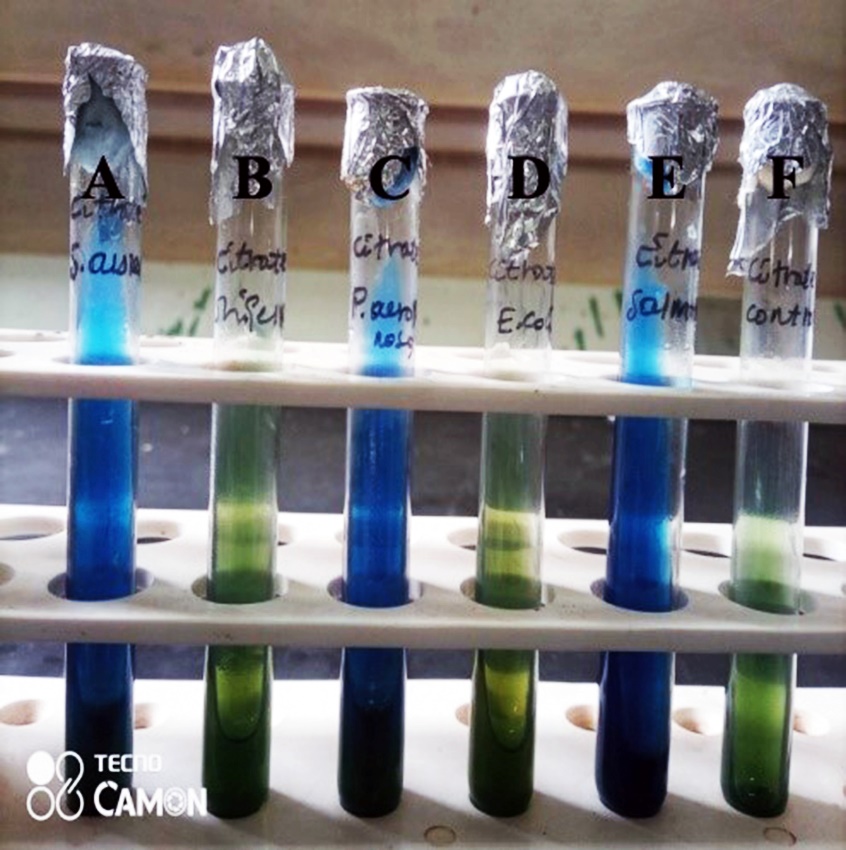


**Figure S4.** Biochemical tests for the identification of bacteria pathogens

**Indole Test: A** - *E. coli* (Positive); B – Control

**Coagulase Test: A** - *S. aureus* (Positive); B – Control


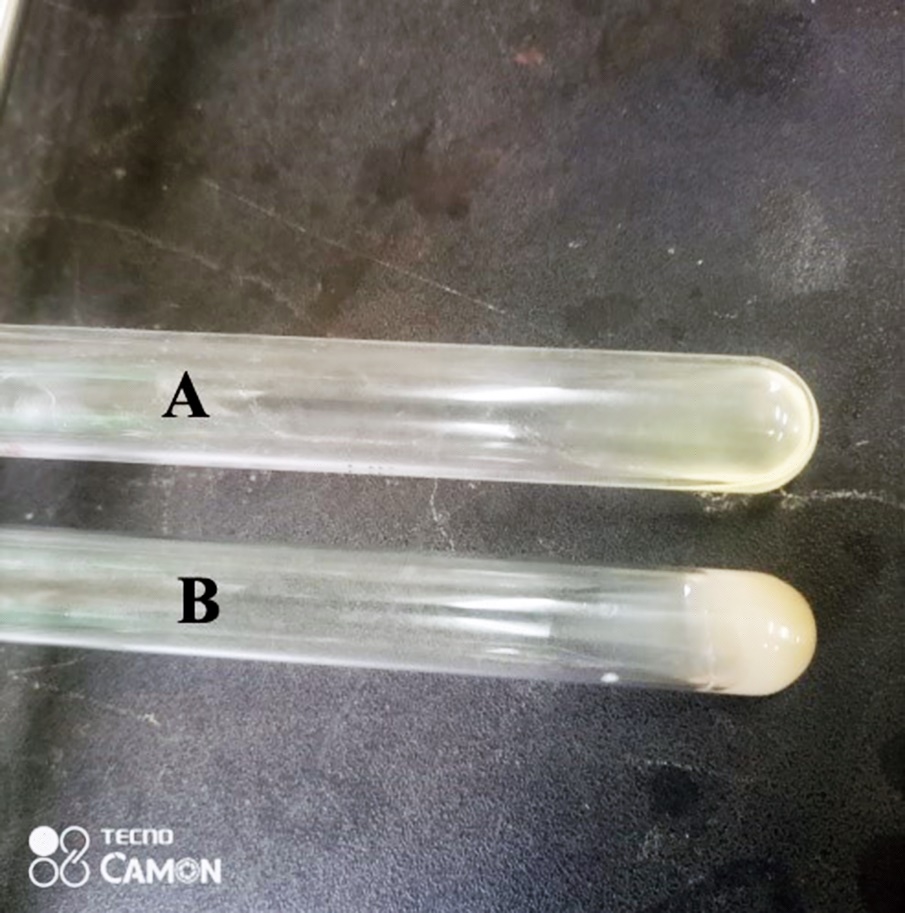

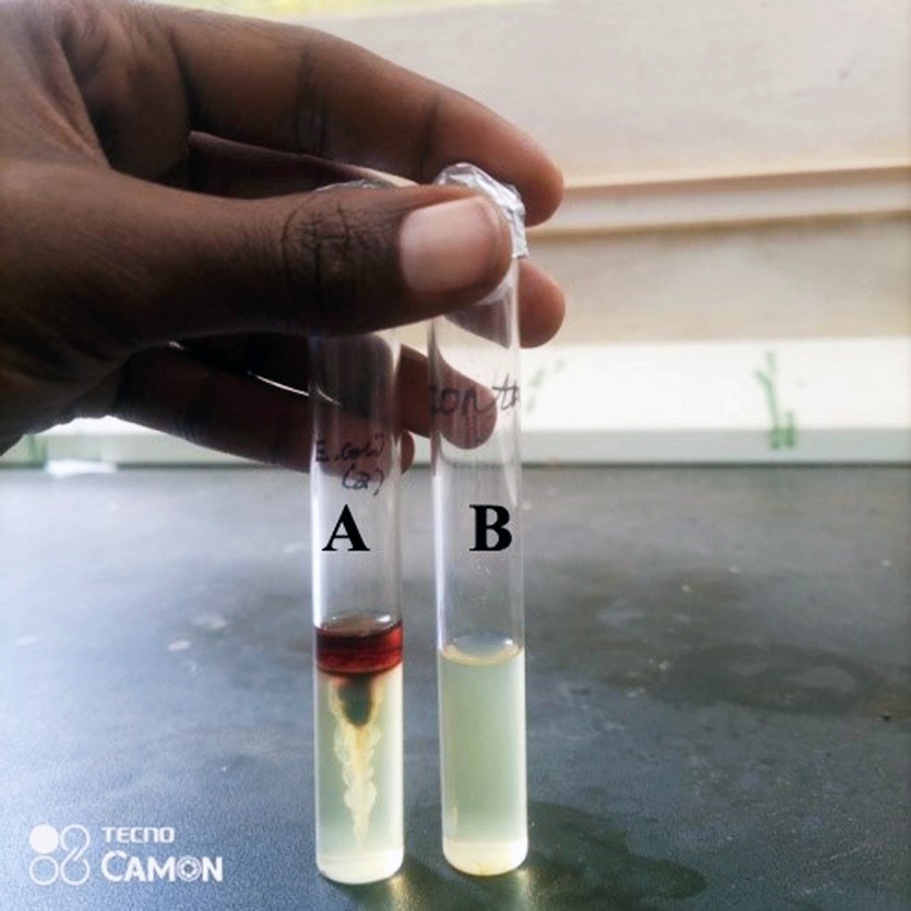


**Figure S5.** Biochemical tests for the identification of bacteria pathogens

**Methyl Red Test:** A – *Salmonella* spp. (Positive); B - *E. coli* (Positive); C – *S. aureus* (Positive); D – *Shigella* spp. (Positive); E – *P. aeruginosa* (Negative); F – Control

**Catalase Test:** A – *Shigella* spp. (Positive); B – *P. aeruginosa* (Positive); C – *S. aureus* (Positive); D - *E. coli* (Positive); E – *Salmonella* spp. (Positive);


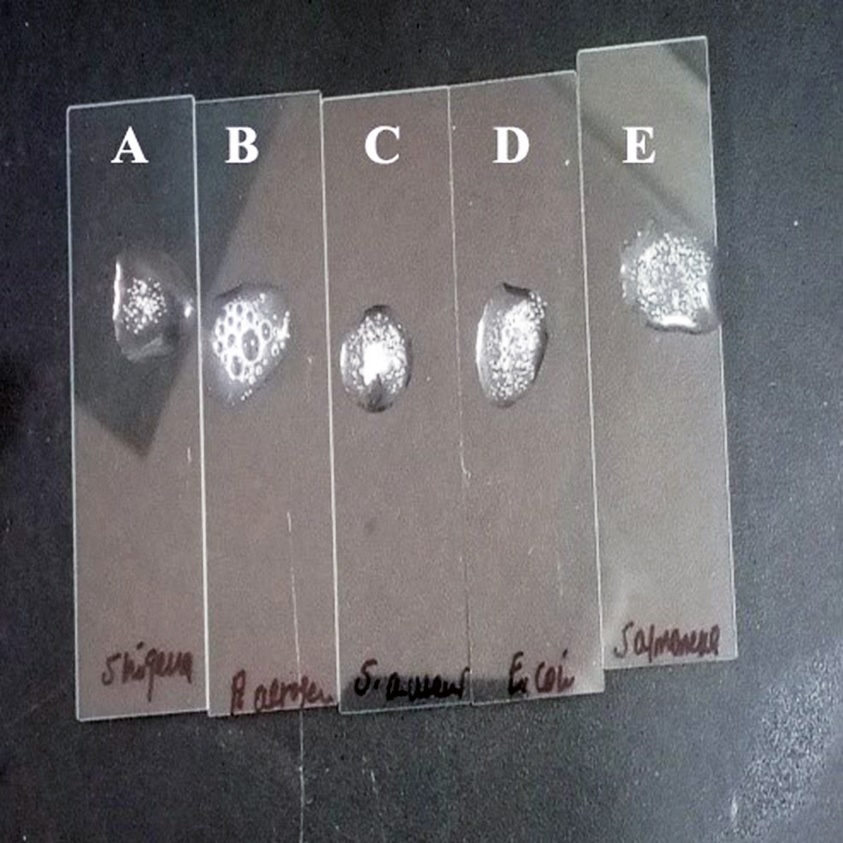

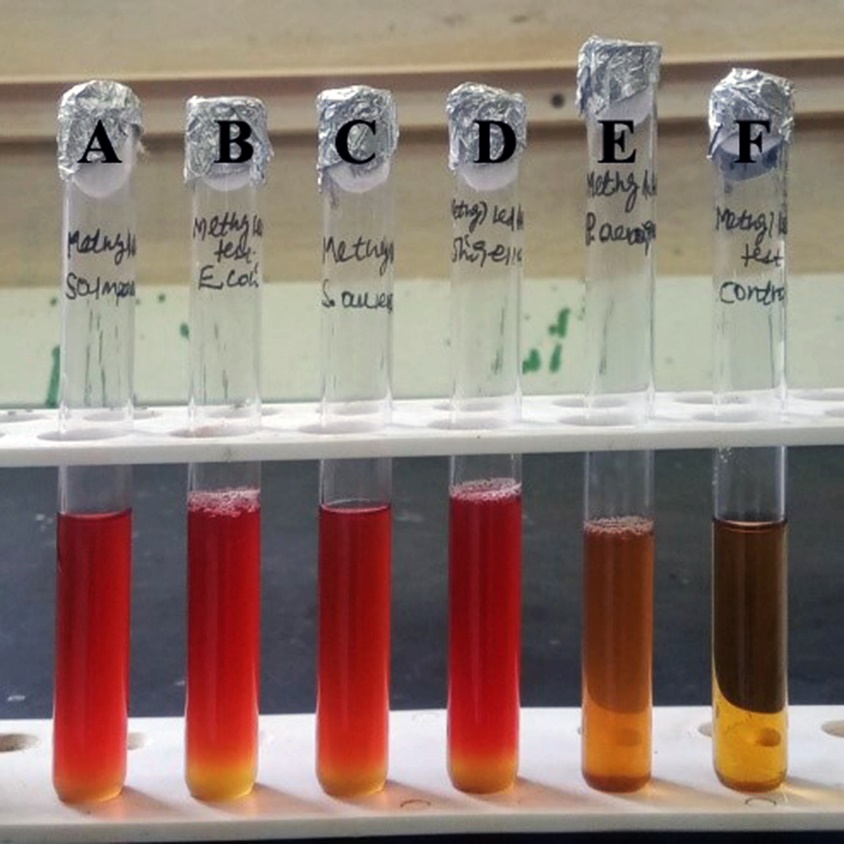


**Figure S6.** Biochemical tests for the identification of bacteria pathogens

# Table S1. Prevalence of bacterial pathogens isolated from the water samples of the Kalte River

| Sites | Id.no | Isolated bacterial population | | | | |
| --- | --- | --- | --- | --- | --- | --- |
|  |  | **E. coli** | ***Salmonella spp.*** | ***Shigella* spp.** | ***S. aureus*** | ***P. aeruginosa*** |
| Dam(S1) | DSS-1 | - | - | - | + | - |
|  | DSS-2 | - | - | - | - | + |
|  | DSS-3 | + | - | - | - | - |
|  | DSS-4 | - | - | - | - | - |
|  | DSS-5 | - | - | - | - | - |
|  | DSS-6 | - | - | - | - | - |
|  | DSS-7 | - | - | - | - | - |
|  | DSS-8 | + | - | + | - | - |
|  | DSS-9 | - | - | - | - | + |
|  | DSS-10 | - | + | - | - | - |
|  | DSS-11 | + | - | - | - | - |
|  | DSS-12 | - | - | - | + | - |
|  | DSS-13 | + | - | - | - | + |
|  | DSS-14 | + | - | - | - | - |
| Ker(S2) | KSS-1 | + | - | - | + | - |
|  | KSS-2 | - | + | - | - | + |
|  | KSS-3 | + | - | - | + | - |
|  | KSS-4 | + | - | + | - | + |
|  | KSS-5 | - | - | - | + | + |
|  | KSS-6 | + | - | - | - | - |
|  | KSS-7 | - | + | - | - | - |
|  | KSS-8 | + | - | - | - | + |
|  | KSS-9 | + | - | + | + | + |
|  | KSS-10 | + | + | - | - | + |
|  | KSS-11 | + | - | - | + | - |
|  | KSS-12 | + | + | + | + | + |
|  | KSS-13 | + | + | - | - | + |
|  | KSS-14 | + | - | - | - | - |
| Gut(S3) | GSS-1 | + | - | - | - | + |
|  | GSS-2 | - | - | - | + | - |
|  | GSS-3 | - | - | - | - | - |
|  | GSS-4 | + | - | - | - | + |
|  | GSS-5 | + | + | - | - | - |
|  | GSS-6 | - | - | + | - | - |
|  | GSS-7 | - | - | - | - | - |
|  | GSS-8 | + | - | - | - | - |
|  | GSS-9 | - | - | - | + | + |
|  | GSS-10 | + | - | - | - | - |
|  | GSS-11 | + | - | + | - | + |
|  | GSS-12 | + | - | - | + | - |
|  | GSS-13 | + | + | - | - | + |
|  | GSS-14 | + | - | - | - | + |

**Legend**: Dam-Damota, Ker-Kera, Gut-Gututo, + = Positive (presence) - = Negative (absence); Id.no -Identification number of sample, DSS-Damota site sample, KSS-Kera site sample; GSS-Gututo site sample
